# Unconjugated Bilirubin Attenuates DSS-Induced Colitis Potentially *via* Enhancement of Bilirubin Reabsorption

**DOI:** 10.3389/fphar.2021.654808

**Published:** 2021-05-20

**Authors:** Chong Zhao, Hongli Huang, Qiuhua Pan, Wenqi Huang, Wu Peng, Haoming Xu, Zhiqiang Feng, Yanlei Du, Yuqiang Nie, Yongjian Zhou

**Affiliations:** ^1^ Department of Gastroenterology, Guangzhou Digestive Disease Center, Guangzhou First People’s Hospital, School of Medicine, South China University of Technology, Guangzhou, China; ^2^ Guangzhou Key Laboratory of Digestive Disease, Guangzhou First People’s Hospital, Guangzhou, China; ^3^ Guangzhou Municipal and Guangdong Provincial Key Laboratory of Protein Modification and Degradation, School of Basic Medical Sciences, Guangzhou Medical University, Guangzhou, China

**Keywords:** ulcerative colitis, bilirubin, antioxidant, enterohepatic cycling, superoxide (O2^·−^)

## Abstract

Studies increasingly show that ulcerative colitis (UC) is a consequence of an imbalance between oxidative stress and antioxidant capacity. Bilirubin exerts an anti-inflammatory effect by scavenging reactive oxygen species (ROS), although the exact mechanism is not completely understood. The aim of this study was to determine the role of serum bilirubin in UC using patient data and a mouse model of dextran sodium sulfate (DSS)-induced colitis. We found that low levels of serum bilirubin correlated to a higher risk of UC in a retrospective case-control population. Pre-treatment with exogenous unconjugated bilirubin (UCB) significantly enhanced colonic bilirubin absorption in mice, and attenuated the DSS-induced body weight loss, colon shortening and histopathological damage. Mechanistically, bilirubin prevented the infiltration of inflammatory cells, and decreased the levels of myeloperoxidase and pro-inflammatory cytokines in the serum and colon. Moreover, bilirubin inhibited ROS and malondialdehyde production, scavenged superoxide anions (O_2_^·−^) from the colon and enhanced the total antioxidant capacity. In conclusion, exogenous UCB attenuated DSS-induced colitis by directly scavenging O_2_^·−^ and enhancing bilirubin reabsorption in the colon via enterohepatic cycling.

## Introduction

Ulcerative colitis (UC) is an idiopathic chronic inflammatory bowel disease primarily involving the colonic mucosa and submucosa (Ungaro et al., 2017). Multiple pro-inflammatory mediators including reactive oxygen species (ROS), cytokines and neutrophils have been implicated in the pathogenesis of UC (Sands, 2015). Studies increasingly show that oxidative stress through reactive oxygen metabolites (ROMs) is the fundamental mechanism of intestinal tissue damage (Simmonds et al., 1992; Barbosa et al., 2003; Aviello and Knaus, 2017). ROS or free radicals are a byproduct of mitochondrial oxidative phosphorylation, and are quenched by the antioxidant system defense under physiological conditions. However, the antioxidant enzymes are usually impaired in pathological conditions (Pisoschi and Pop, 2015), resulting in excessive production of ROS that correlates with the pathogenesis and development of inflammatory diseases. Studies show that activated neutrophils in the intestinal mucosa produce high levels of ROS, which can induce oxidative stress (Naito et al., 2007a; Naito et al., 2007b). The predominant intracellular ROS are superoxide anions (O_2_^·−^) that are generated by the reduction of oxygen. Massive infiltration and activation of neutrophils to injured tissues significantly increase the *in situ* O _2_^**.-**^ levels, leading to the formation of other ROMs such as hydrogen peroxide (H_2_O_2_), hydroxyl radicals (OH^−^) and hypochlorous acid (HOCl) (Bergamini et al., 2004; Murphy, 2009). The acute oxidative damage in the colonic mucosa triggers an inflammatory response, and the resulting tissue damage further exacerbates ROS and ROMs production, eventually forming a positive feedback loop that renders the antioxidant defense ineffective (Roessner et al., 2008). Elevated levels of oxidized proteins have been observed in the ulcerative colonic mucosa, and the drugs used to treat UC are effective ROM scavengers. This strongly suggests that ROS play a crucial role in the pathophysiology of UC (Reifen et al., 2004; Xu et al., 2004), and scavenging excess ROS is critical for mitigating intestinal inflammation.

Unconjugated bilirubin (UCB) is an antioxidant tetrapyrrole derived from the catabolism of heme (Salomone et al., 2013). The unreduced UCB in the gut lumen is partly absorbed by the enterocytes and transported to the liver via the portal venous blood. This enterohepatic circulation of UCB contributes to bilirubin homeostasis and maintains normal levels of UCB in the plasma (Hamoud et al., 2018). UC patients with jaundice exhibit milder symptoms compared to those with normal bilirubin levels (Papatheodoridis et al., 1998). Likewise, patients with Gilbert syndrome who have elevated UCB levels due to defective UDP glucuronosyl transferase 1 (UGT1A1) activity are less likely to suffer from UC, further supporting the protective effect of UCB against intestinal inflammation (de Vries et al., 2012; Lenicek et al., 2014). Although the role of serum bilirubin in UC patients has been evaluated, the mechanisms underlying the pathological association are not completely understood. There is evidence indicating that exogenous bilirubin mitigates gut inflammation in an animal model of inflammatory bowel disease (IBD) (Zucker et al., 2015; Longhi et al., 2017). However, little is known regarding the potential antioxidant effects of bilirubin against colitis. The aim of this study was to evaluate the effect of intestinal UCB absorption on colonic inflammation and oxidative stress in a dextran sodium sulfate (DSS)-induced mouse model of colitis.

## Materials and Methods

### Reagents

Unconjugated bilirubin (bilirubin IXα) was obtained from Sigma-Aldrich (St. Louis, MO, United States), and the stock solution was freshly prepared in 0.1 M potassium phosphate (pH 12) as previously described (Huang et al., 2017). DSS (36–50 kDa) was obtained from MP Biomedical (Solon, OH, United States).

### Patients and Controls

Eighty-nine UC patients that were evaluated at the Department of Gastroenterology and Gastrointestinal Surgery, Guangzhou First People’s Hospital, between January 2017 and December 2019 were recruited. UC was diagnosed in accordance with the guidelines of the European Crohn’s and Colitis Organization (ECCO). Patients with Gilbert syndrome and primary sclerosing cholangitis, or any other autoimmune disease that might influence serum total bilirubin (sTB) levels, were excluded from the study. Healthy subjects who visited the hospital for routine physical examination during the same period were included in the control group. The clinical and laboratory data were retrieved from the medical record systems, and sTB levels were measured by standard methods. The study was approved by the Institutional Review Board and Ethics Committee, and all participants signed the informed consent form.

### 2.3 Mouse Model of DSS-Induced Colitis

Male C57BL/6 mice aged 8–12 weeks (weight approximately 25 g) were purchased from the Guangdong Laboratory Animal Monitoring Institute, and housed under 12 h light/dark cycles with ad libitum access to food and water. Colitis was induced in the adult mice by administering 3% DSS (w/v) in the drinking water. The control group received filtered water without DSS. Twenty-four hours before commencing DSS treatment, the animals were given daily intraperitoneal (ip) injections of bilirubin (30 mg/kg) or an equivalent volume of the potassium phosphate for indicated days. All animal experiments were approved by the Institutional Animal Care and Use Committee of South China University of Technology.

### Assessment of Disease Activity

The mice were weighed and examined for signs of colitis on a daily basis. The disease activity index (DAI) was graded as follows: a) Body weight loss: 0—none, 1—1–5%, 2—5–10%, 3—10–15%, 4—>15%; b) stool consistency: 0—normal, 2—loose stools, 4—diarrhea; c) blood in stool: 0—negative, 2—positive, 4—gross rectal bleeding. The mean scores were calculated and recorded as the DAI. After the 7-day treatment, the animals were euthanized by CO_2_ inhalation, and blood was immediately drawn by cardiac puncture. The small and large intestines were resected, and the colon length was measured.

### Histological Examination

Longitudinal sections of proximal and distal colon were fixed in 4% paraformaldehyde and stained with hematoxylin and eosin as per standard protocols. The specimens were analyzed independently by three investigators blinded to the grouping. At least 10 low-power non-overlapping fields were examined per section, and histologically scored on the basis of the following parameters: a) severity of inflammation: 0—no inflammation. 1—mild, 2—moderate and 3—severe; b) depth of inflammatory involvement: 0 - no inflammation, 1—mucosa, 2—mucosa and submucosa, and 3—transmural; c) crypt damage: 0—intact crypts, 1—loss of the basal one third, 2—loss of the basal two thirds, 3—entire crypt loss but intact epithelial surface, and 4—entire crypt loss and erosion of epithelial surface. The scores of all three parameters were added, and the maximum score was 40.

### Enzyme-Linked Immunosorbent Assay

The colon pieces were homogenized in lysis buffer, and then centrifuged at 12,000 × *g* at 4°C for 30 min. The protein content in the supernatant was determined using bicinchoninic acid (BCA)™ protein assay kit (Thermo, MA, United States). IL-1β, IL-6, TNF-α, IFN-γ and MPO levels were measured by ELISA using specific kits (Abcom; Cambridge, United States) as per the manufacturer’s instructions, and expressed as pg/mg or U/g (MPO).

### Measurement of Malondialdehyde

Colonic tissues were homogenized in ice-cold lysis buffer, and then centrifuged at 12,000 × *g* at 4°C for 30 min. Levels of malondialdehyde (MDA) in colon tissues were measured by the kits according to the manufacturer’s instructions from Beyotime Institute of Biotechnology (Haimen, China). The total protein content was determined by a BCA™ protein kit (Thermo, MA, United States).

### Measurement of Reactive Oxygen Species Levels

ROS levels were measured using the fluorescent 2, 7-dichlorofluorescein-diacetate probe (DCFH-DA, Beyotime Institute of Biotechnology, China). The colon specimens were homogenized with PBS into a single-cell suspension. The cells were harvested and incubated with DCFH-DA for 30 min at 37°C in the dark. The samples were acquired by flow cytometry, and the fluorescence intensity was measured.

### Measurement of Total Antioxidant Capacity

Colonic tissues were homogenized in cold PBS. The antioxidant activity in the supernatant was evaluated using a specific kit from Beyotime Institute of Biotechnology (Haimen, China) according to the manufacturer’s instructions.

### Dihydroethidium Staining for Superoxide Anions

The frozen colon tissues were embedded in OCT and cut into 8 μm sections. After staining with 30 μM DHE (Invitrogen Molecular Probes) for 10 min at 37°C, the sections were counterstained with Vectashield containing DAPI (Vector Laboratories, Inc.). The slides were observed with a Zeiss 510 upright confocal microscope, and the fluorescence intensity of at least 100 nuclei per sample was scored in at least three mice.

### HPLC/Electrospray Ionization Tandem Mass Spectrometry

The bilirubin levels in serum and colon homogenates were measured by ion-pair extraction using 0.1 mol/L methanolic di-*n*-octylamine acetate. Briefly, the supernatants were injected into the liquid chromatography mass spectrometer (LC-MS/MS) system was equipped with an Agilent 1200 liquid chromatograph and a 6410B triple quadrupole mass spectrometer with an ESI source. Data were analyzed using the MassHunter software (Agilent Corporation, Lexington, MA, United States). Chromatography was performed on a Poroshell 120 EC-C18 2.1 μm 30 × 50 mm column (Agilent).

### Measurement of Superoxide Anions

Colonic tissues were collected immediately and homogenized in ice-cold PBS, and centrifugation at 12,000 × *g* at 4°C for 30 min and kept at −80°C until they were assayed. Superoxide anion (O_2_^·−^) production was detected using a LumiMax Superoxide Anion Detection kit (Agilent Technologies, La Jolla, CA, United States) according to the manufacturer’s protocol. Briefly, 50 μg colon proteins were suspended in 100 μl assay medium and then mixed with 100 μl detection reagent containing 0.2 mM luminol and 0.25 mM enhancer. The luminescence was measured using a luminometer, and the O_2_^·−^ content was expressed as relative light units (RLU)/μg protein/min. The total protein content was determined by a BCA™ protein kit (Thermo, MA, United States).

### Immunohistochemistry

The colon tissues were fixed in 4% paraformaldehyde, embedded in paraffin and sectioned as previously described (Lu et al., 2011). The sections were immunostained using MaxVision kit (Maixin Biol, Fuzhou, Fujian, China) according to the manufacturer’s instructions. The primary bilirubin was diluted 1:100 in blocking solution. Color was developed using 0.05% diaminobenzidine and 0.03% H_2_O_2_ in 50 mM Tris–HCl (pH 7.6), and the sections were counterstained with hematoxylin. Pre-immune rabbit serum was used as the negative control. The anti-bilirubin antibody was purchased from LifeSpan BioSciences (Seattle, WA, United States).

### Statistical Analyses

All statistical analyses were performed using GraphPad Prism 5 (GraphPad Software, United States) and SPSS version 21.0 (IBM SPSS Statistics, United States). For the clinical study, non-normally distributed variables are expressed as median (interquartile range) and categorical variables as absolute numbers. For the animal study, the data are presented as mean ± standard error of the mean (SEM). Differences between groups were determined using one-way analysis of variance followed by Tamhane’s multiple comparisons post-hoc tests using SPSS. *p* < 0.05 was considered statistically significant.

## Results

### Total Serum Bilirubin Levels Are Reduced In Inflammatory Bowel Disease Patients

A total of 89 UC patients (average age - 43.37 years, male/female ratio - 48/) and 95 healthy controls (average age - 38.76 years, male/female ratio - 50/39) were included in this study. The electronic medical records of the case and control populations were screened for total serum bilirubin levels. As shown in Table 1, the UC patients had significantly lower median serum bilirubin levels compared to the controls (9.8 *vs.* 12.5 μM, *p* < 0.0001). When stratified by age and sex, the reduction in serum bilirubin levels was more pronounced in males compared to females, and in the middle-aged patients (30–50 years old).

**TABLE 1:** Total serum bilirubin levels are reduced in IBD patients.

Group	Control (*n* = 95)	*p* value	UC (*n* = 89)	*p* value
	Serum bilirubin (μmol/L)		Serum bilirubin (μmol/L)	
Total	12.5 (9.1–14.9)	—	9.8 (8.55–13.15)	0.0023
Sex				
Male	13.2 (9.05–15.38) (*n* = 48)	0.2138	10.15 (8.35–13.38) (*n* = 50)	0.4177
Female	12.0 (9.1–13.9) (*n* = 47)		9.8 (8.7–11.4) (*n* = 39)	
Age				
<30	11.5 (8.4–13.35) (*n* = 29)	0.1115	9.0 (6.75–11.75) (*n* = 21)	0.1813
30–50	13.25 (8.77–15.4) (*n* = 40)		10.5 (7.76–14.05) (*n* = 33)	
>50	12.35 (9.95–15.15) (*n* = 26)		9.8 (8.7–12.7) (*n* = 35)	

### Exogenous Unconjugated Bilirubin Mitigates the Symptoms of Dextran Sodium Sulfate-Induced Colitis

**FIGURE 1:**
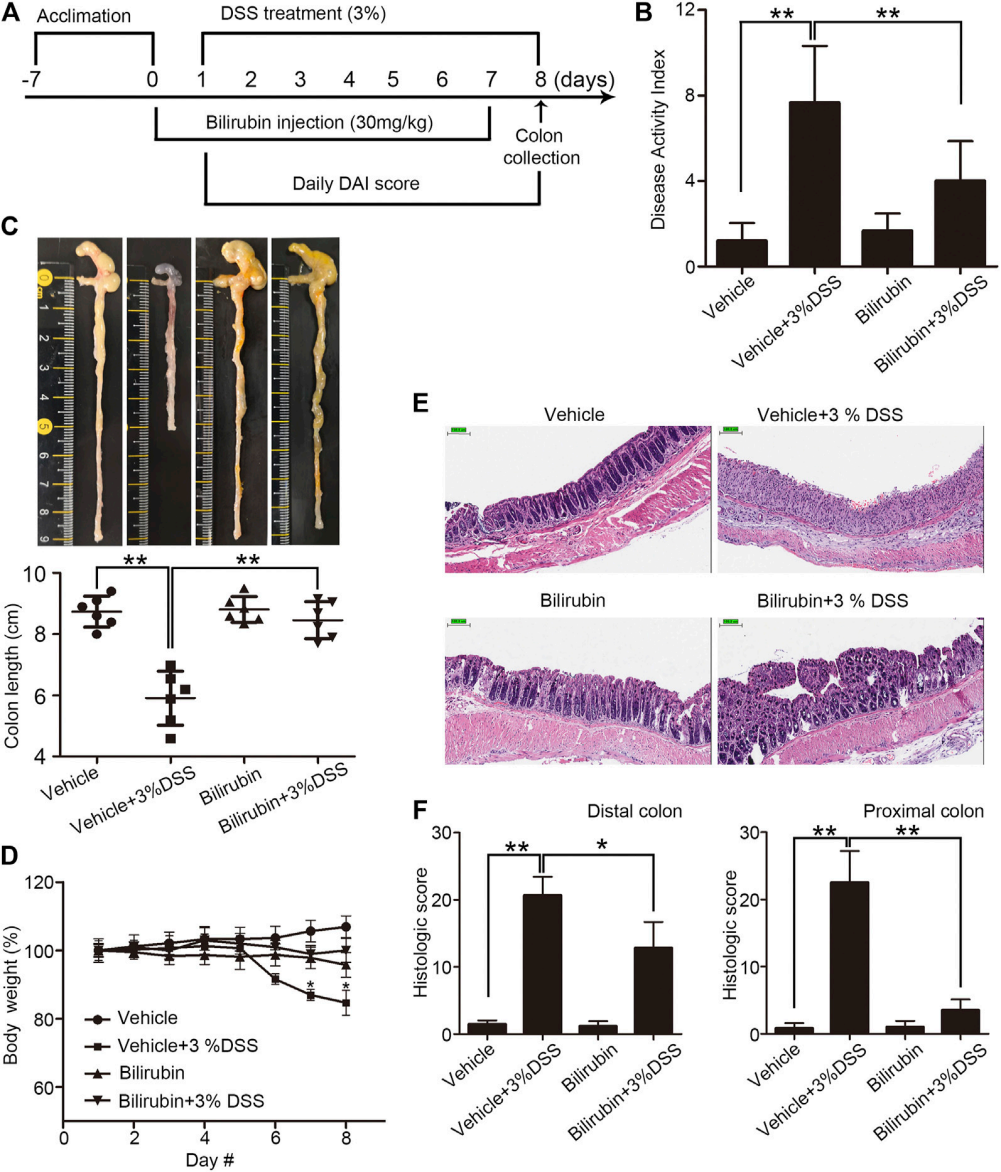
Effect of bilirubin on DSS-induced colitis. **(A)** Experimental design. **(B)** Disease activity index scores (DAI) in the indicated mice. Data are expressed as mean ± SEM (*n* = 6); **p < 0.01. **(C)** Macroscopic appearance **(top)** and the length of colons **(bottom)** in the indicated groups. Data are expressed as mean ± SEM (*n* = 6); **p < 0.01. **(D)** Body weight of mice during the experiment duration. Data were normalized as percentage of basal body weight. *p < 0.05 *vs.* the colitis group. **(E)** Representative images of HE-stained colon tissue sections in the indicated groups. Bar = 100 μm. **(F)** Mean histological scores of distal (left) and proximal (right) colon in the indicated groups. Data are expressed as mean ± SEM (*n* = 6); *p < 0.05 and **p < 0.01.

Given the reduced bilirubin levels in UC patients, we assessed the potential therapeutic effects of exogenous UCB in a mouse model of DSS-induced colitis. The animals received UCB for 24 h prior to colitis induction (Figure 1A). As shown in Figures 1B–D, DSS increased DAI, and reduced the colon length and body weight. In contrast, pre-treatment with UCB improved the DAI, and restored colon length and body weight compared to the vehicle-treated DSS mice. Consistent with the clinical findings, UCB also reduced the infiltration of inflammatory cells in the colon mucosa, and alleviated the mucosal/epithelial damage as per the histological scores (Figure 1E), although the effect was less pronounced in the distal compared to the proximal colon (Figure 1F). In addition, UCB had no impact on any of the above parameters in healthy mice. Taken together, UCB alleviated DSS-induced inflammation and tissue damage.

### Unconjugated Bilirubin Reduced Colonic Inflammation in the Dextran Sodium Sulfate-Treated Mice

**FIGURE 2:**
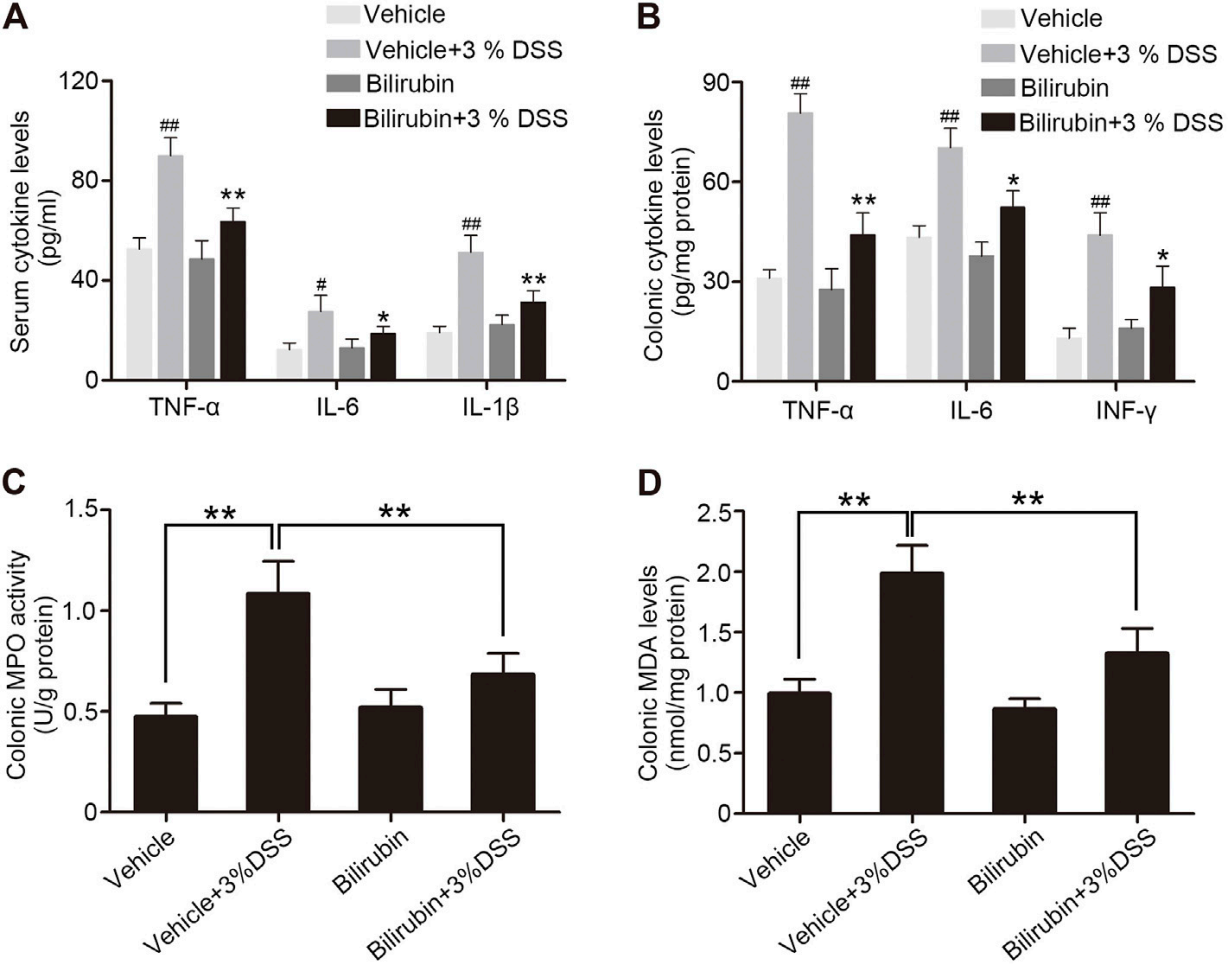
UCB alleviates inflammation in the colon of DSS-treated mice. **(A)** Serum levels of TNF-α, IL-6 and IL-1β in the indicated groups. Data are expressed as mean ± SEM (*n* = 6). *p < 0.05 and **p < 0.01 *vs.* the colitis group. ^#^ *p* < 0.05 and ^##^ *p* < 0.01 *vs.* the vehicle group. **(B)** TNF-α, IL-6 and IFN-γ levels in the colonic homogenates from the indicated groups. Data are expressed as mean ± SEM (*n* = 6). *p < 0.05 and **p < 0.01 *vs.* the colitis group. ^##^ *p* < 0.01 *vs.* the vehicle group. **(C, D)** Colonic MPO activity and MDA levels in the indicated groups. Data are expressed as mean ± SEM (*n* = 6); **p < 0.01.

The severity of acute colitis often correlates with increased levels of pro-inflammatory cytokines. The mice pre-treated with UCB had significantly lower levels of TNF-α, IL-6, INF-γ and IL-1β in the serum as well as colonic tissues compared to the PBS-treated group (Figures 2A,B). In addition, the colonic myeloperoxidase (MPO) activity, a measure of neutrophil infiltration, was considerably increased in the untreated model group and decreased markedly in the mice pre-treated with UCB (Figure 2C). Finally, high levels of MDA were detected in the colon of untreated mice with colitis (Figure 2D), indicating significant oxidative injury. Pre-treatment with UCB reduced the MDA content to near baseline levels. Taken together, UCB has a potent anti-inflammatory effect in colitis.

### Exogenous Unconjugated Bilirubin Increased Bilirubin Levels in the Plasma and Colon of Dextran Sodium Sulfate-Treated Mice

**FIGURE 3:**
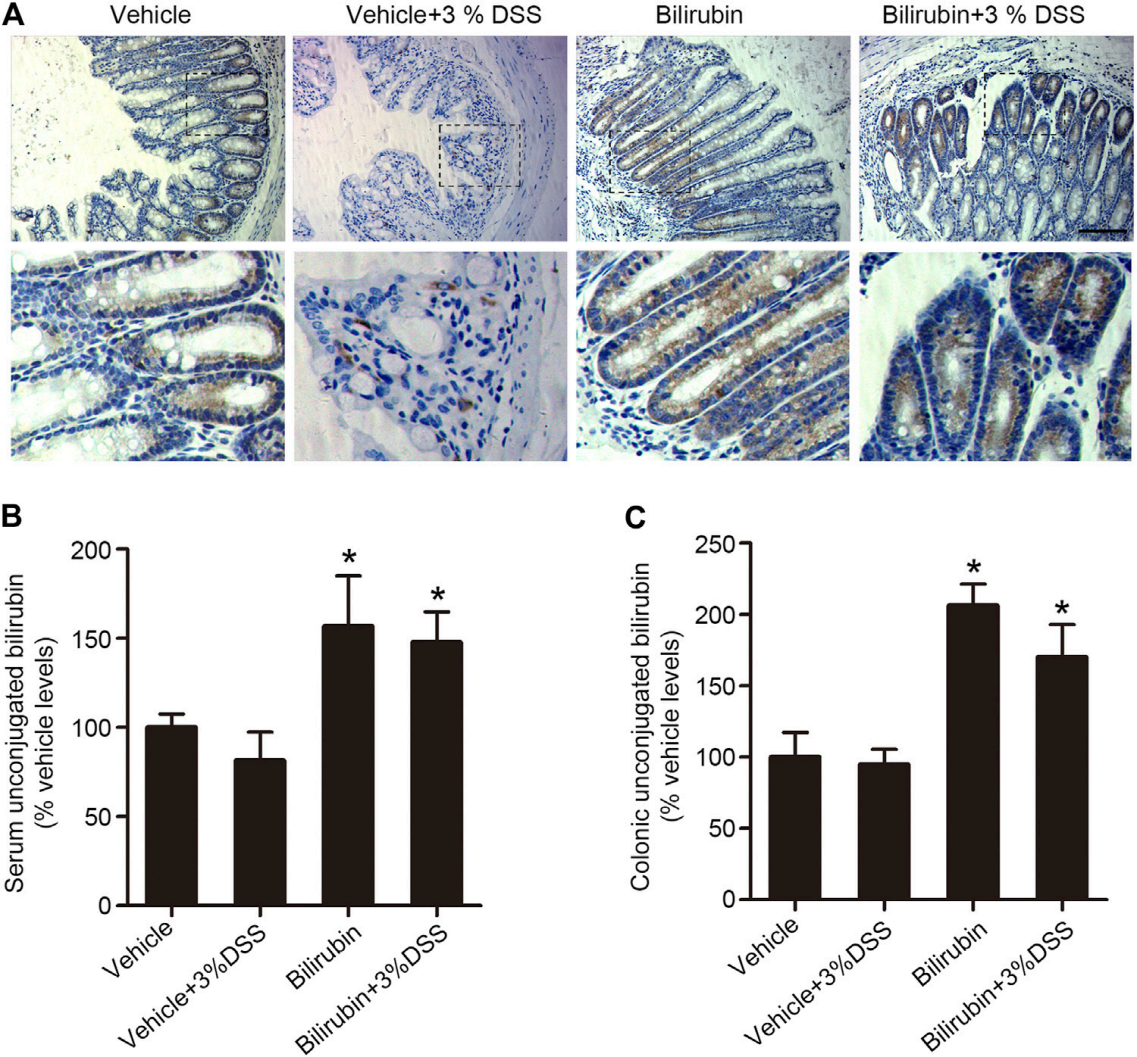
UCB increased plasma and proximal colonic bilirubin levels in the DSS-treated mice. **(A)** Representative images of proximal colon tissues immune-stained for bilirubin in the indicated groups. Bar = 100 μm. **(B, C)** UCB levels in the plasma **(B)** and cecal/proximal colon homogenates **(C)** as measured by LC-MS/MS. Data are expressed as percentage of controls (vehicle). Each bar represents the mean ± SEM (*n* = 6); *p < 0.05 *vs.* the vehicle group.

The gut-liver reabsorption of UCB is essential for normal gut function, and is a potential factor in the development of intestinal diseases. UCB is the β-glucuronidase hydrolysis product of bilirubin glucuronides, and can be absorbed passively from both the large and small intestines. In the human digestive system, the cecum and proximal colon are more acidic compared to the other parts of the intestinal bowel, and therefore more favorable sites for the absorption of diacidic UCB. To determine whether reabsorption of UCB plays a role in DSS-induced colitis, we assessed its localization *in situ*. Bilirubin was mainly localized in the proximal colon tissues of mice pre-treated with UCB, with considerable enrichment in the intestinal epithelial cells (Figure 3A). UCB levels were also measured using LC-MS/MS, which showed that mice pre-treated with UCB had respectively 2-fold and 1.5-fold higher UCB in their proximal colon tissues and serum compared to the vehicle-treated controls (Figures 3B,C). Taken together, UCB supplementation increased its reabsorption from the colonic contents.

### Unconjugated Bilirubin Alleviated Oxidative Stress in the Inflamed Colon of Dextran Sodium Sulfate-Treated Mice

**FIGURE 4:**
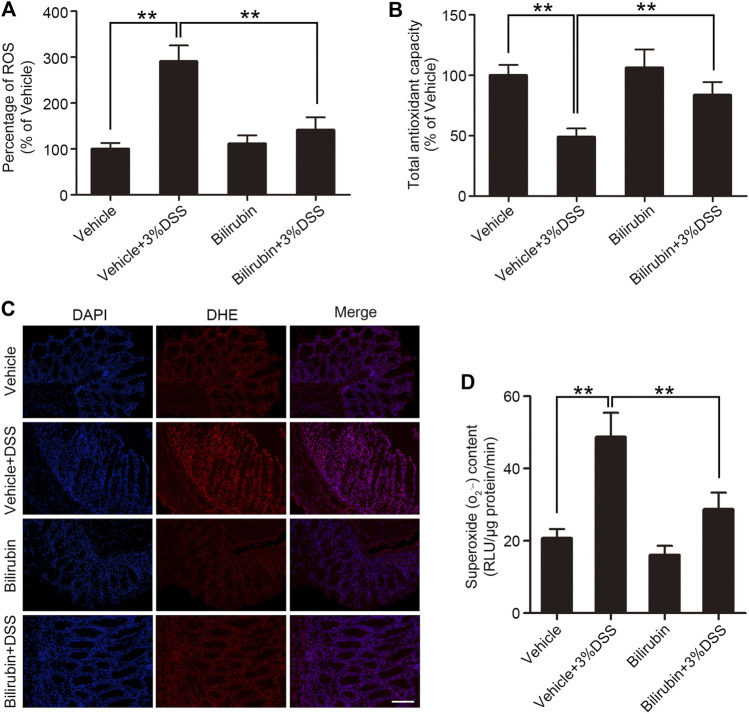
UCB reduced colonic oxidative stress in mice with DSS-induced colitis. **(A)** Relative ROS levels **(A)** and **(B)** total antioxidant capacity (TAC) in the indicated groups. Data are expressed as percentage of controls (vehicle). Each bar represents the mean ± SEM (*n* = 6); *p < 0.05 and **p < 0.01. **(C)** Representative images of DHE-stained colon tissues from the indicated groups. Bar = 100 μm. **(D)** Superoxide anion (O_2_^·−^) levels in the indicated groups. Data are expressed as mean ± SEM (*n* = 6). **p < 0.01.

Overproduction of ROS is involved in the progression and pathogenesis of various acute and chronic inflammatory diseases. There is evidence suggesting that bilirubin has antioxidant activity, especially against O_2_^·−^. Therefore, we also evaluated ROS levels in colon tissues using the fluorescent DCFH-DA probe. UCB administration decreased ROS generation in the colon tissues of DSS-treated mice (Figure 4A). In addition, UCB also restored TAC levels that was markedly reduced in the DSS-treated mice to baseline (Figure 4B). The O_2_^·−^ level in colon tissues was next measured by DHE staining, which revealed increased O_2_^·−^ generation in the vehicle group which declined upon UCB pre-treatment (Figure 4C). Likewise, the specific detection kit also indicated a significant accumulation of O_2_^·−^ in the colitis model, which was markedly reduced by UCB pre-treatment (Figure 4D). To summarize, exogenous UCB alleviated oxidative stress in the colitis model by directly scavenging O_2_^·−^ from the inflamed colon tissues.

## Discussion

Multiple clinical studies have reported that bilirubin levels correlate negatively with the risk of IBD (Lenicek et al., 2014; Tian et al., 2018; Zhao et al., 2019). Consistent with this, we detected significantly lower serum levels of total bilirubin in UC patients compared to healthy individuals. This suggested that UC patients may have abnormal bilirubin metabolism, resulting in reduce total serum bilirubin. To explore the potential therapeutic effect of UCB on gut inflammation, we established the DSS-induced colitis model that closely mimics human IBD (Chassaing et al., 2014). Exogenous UCB attenuated the acute intestinal injury, weight loss and colon shortening induced by DSS administration. Mechanistically, UCB prevented DSS-induced inflammation by decreasing neutrophil infiltration into the colon tissues, and the *in situ* production of pro-inflammatory cytokines such as IL-1β, IL-6, IFN-γ and TNF-α. Consistent with this, UCB treatment also decreased the activity of myeloperoxidase (MPO), which is released by the neutrophils during an inflammatory response (Masoodi et al., 2012).

Experimental and clinical evidence indicate that ROS overproduction plays an important role in the development and progression of IBD, and the mucosal ROS levels are 10-100 fold higher in IBD patients compared to healthy controls (Sedghi et al., 1993; Zhu and Li, 2012). In the DSS colitis model as well, the colon tissues showed accumulation of the lipid peroxidation product MDA (Gueraud et al., 2010), along with a distinct reduction in the total antioxidant capacity (TAC) that reflects the sum of all endogenous and exogenous antioxidants (Serafini and Del Rio, 2004). UCB pre-treatment restored the colonic TAC to nearly normal control levels, indicating that its protective effects on DSS-induced colitis are mediated via reduction of oxidative stress. Bilirubin inhibits NADPH oxidase, which is the primary source of O_2_^·−^ that causes oxidative damage and inflammation in the intestinal mucosa (Lanone et al., 2005). Therefore, lower levels of bilirubin in UC patients may increase systemic and localized oxidative stress (Koutroubakis et al., 2004). A recent study also showed that bilirubin protected neuronal cells against free radical damage by scavenging O_2_^·−^ (Vasavda et al., 2019). Consistent with this, we found that UCB directly scavenged O_2_^·−^ from the inflamed colons of DSS-treated mice.

Although exogenous bilirubin mitigated inflammation in an animal model of IBD via systemic antioxidant action, little is known regarding its effects on local lesions. Therefore, we also evaluated whether intestinal reabsorption of UCB enhanced the local accumulation of bilirubin in the DSS-induced colitis model by immunostaining and LC-MS/MS analysis. An apparent increase in UCB was observed in the epithelial cells of the proximal colon tissues in mice treated with UCB, suggesting that bilirubin can be absorbed from colonic contents. Since the intraluminal pH of the proximal colon is mildly acidic as opposed to the highly acidic stomach and neutral distal colon, it is the favored site for absorption of diacidic UCB (Brink et al., 1996; Evans et al., 1988). Accordingly, we found that the protective effect of bilirubin was more pronounced in the proximal colon compared to the distal colon, which is also consistent with another study on the DSS-induced colitis mice (Zucker et al., 2015). Taken together, we hypothesize that bilirubin is a physiological antioxidant that acts locally at the primary site of colitis, and exogenous UCB enhances its reabsorption in the proximal colon.

The enterohepatic cycling of bilirubin is a common occurrence in newborns, and can reappear in adult life. It occurs exclusively with the unconjugated form (Berk, 1994) since the enterocytes prevent passive transport of conjugated bilirubin on account of its bulky size and polarity. The bacterial β-glucuronidase hydrolyzes conjugated bilirubin glucuronide to facilitate its reabsorption in the distal small bowel and colon (Tiribelli and Ostrow, 2005). We found that exogenous UCB supplementation increased its systemic levels, which may promote enterohepatic reabsorption of bilirubin. Wang et al. reported that the total bilirubin concentration in rat serum peaked to 4-fold higher levels than the baseline within 60 min of a single intraperitoneal injection of bilirubin at the same dose used in our experiment (Wang et al., 2004). A recent study showed that bacterial β-glucuronidase inhibited DSS-induced colitis in mice (He et al., 2019), which correlates with another study showing that administering β-glucuronidase from extraintestinal sources increased bilirubin deconjugation in the intestine (Vitek and Carey, 2003). The β-glucuronidases in the human intestinal tract are produced by the resident bacterial flora, especially Enterobacteriaceae, Ruminococcaceae and Clostridiaceae (Dabek et al., 2008). Interestingly, these bacteria have also been implicated as potential drivers of IBD pathogenesis (Kostic et al., 2014; Png et al., 2010). Changes in the β-glucuronidase-producing bacteria may alter bilirubin metabolism, resulting in enhanced reabsorption. Therefore, the relationship between bilirubin homeostasis and IBD-associated dysbiosis requires further investigation.

To summarize, exogenous UCB alleviated DSS-induced colitis by directly scavenging O_2_^·−^ and enhancing enterohepatic cycling and passive colonic reabsorption of bilirubin. However, the clinical application of UCB is limited due to its insolubility in water and potential toxicity in various tissues. Nanoparticle carriers can increase the solubility of bilirubin and minimize tissue toxicity through targeted delivery. Bilirubin-nanomedicine has shown encouraging results in murine modeling colitis, and will be our next focus.

## Data Availability

The original contributions presented in the study are included in the article/Supplementary Material, further inquiries can be directed to the corresponding authors.

## References

[B1] Aviello G. Knaus U. (2017). ROS in Gastrointestinal Inflammation: Rescue or Sabotage? Br. J. Pharmacol. 174, 1704–1718. 10.1111/bph.13428 26758851 PMC5446568

[B2] Barbosa D. S. Cecchini R. El Kadri M. Z. Rodríguez M. A. M. Burini R. C. Dichi I. (2003). Decreased Oxidative Stress in Patients with Ulcerative Colitis Supplemented with Fish Oil ω-3 Fatty Acids. Nutrition 19, 837–842. 10.1016/s0899-9007(03)00162-x 14559317

[B3] Bergamini C. Gambetti S. Dondi A. Cervellati C. (2004). Oxygen, Reactive Oxygen Species and Tissue Damage. Cpd 10, 1611–1626. 10.2174/1381612043384664 15134560

[B4] Berk P. (1994). Foreword. Semin. Liver Dis. 14, 321–322. 10.1055/s-2007-1007321 7855625

[B5] Brink M. Mendez-Sanchez N. Carey M. (1996). Bilirubin Cycles Enterohepatically after Ileal Resection in the Rat. Gastroenterol. 110, 1945–1957. 10.1053/gast.1996.v110.pm8964422 8964422

[B6] Chassaing B. Aitken J. D. Malleshappa M. Vijay-Kumar M. (2014). Dextran Sulfate Sodium (DSS)-induced Colitis in Mice. Curr. Protoc. Immunol. 104, 15 25 11–15 25 14. 10.1002/0471142735.im1525s104 24510619 PMC3980572

[B7] Dabek M. McCrae S. I. Stevens V. J. Duncan S. H. Louis P. (2008). Distribution of Î²-Glucosidase and Î²-Glucuronidase Activity and of Î²-Glucuronidase Gene Gus in Human Colonic Bacteria. FEMS Microbiol. Ecol. 66, 487–495. 10.1111/j.1574-6941.2008.00520.x 18537837

[B8] de Vries H. S. Te Morsche R. H. M. Jenniskens K. Peters W. H. M. de Jong D. J. (2012). A Functional Polymorphism in UGT1A1 Related to Hyperbilirubinemia Is Associated with a Decreased Risk for Crohn's Disease. J. Crohn's Colitis 6, 597–602. 10.1016/j.crohns.2011.11.010 22398043

[B9] Evans D. F. Pye G. Bramley R. Clark A. G. Dyson T. J. Hardcastle J. D. (1988). Measurement of Gastrointestinal pH Profiles in normal Ambulant Human Subjects. Gut 29, 1035–1041. 10.1136/gut.29.8.1035 3410329 PMC1433896

[B10] Guéraud F. Atalay M. Bresgen N. Cipak A. Eckl P. M. Huc L. (2010). Chemistry and Biochemistry of Lipid Peroxidation Products. Free Radic. Res. 44, 1098–1124. 10.3109/10715762.2010.498477 20836659

[B11] Hamoud A.-R. Weaver L. Stec D. E. Hinds T. D. Jr. (2018). Bilirubin in the Liver-Gut Signaling Axis. Trends Endocrinol. Metab. 29, 140–150. 10.1016/j.tem.2018.01.002 29409713 PMC5831340

[B12] He Y. Yu H. Ge Y. Li X. Jiang M. Liu Y. (2019). Bacterial β-glucuronidase Alleviates Dextran Sulfate Sodium-Induced Colitis in Mice: A Possible Crucial New Diagnostic and Therapeutic Target for Inflammatory Bowel Disease. Biochem. biophysical Res. Commun. 513, 426–433. 10.1016/j.bbrc.2019.03.196 30967260

[B13] Huang H. Guo M. Liu N. Zhao C. Chen H. Wang X. (2017). Bilirubin Neurotoxicity Is Associated with Proteasome Inhibition. Cell Death Dis 8, e2877. 10.1038/cddis.2017.274 28617443 PMC5520929

[B14] Kostic A. D. Xavier R. J. Gevers D. (2014). The Microbiome in Inflammatory Bowel Disease: Current Status and the Future Ahead. Gastroenterol. 146, 1489–1499. 10.1053/j.gastro.2014.02.009 PMC4034132 24560869

[B15] Koutroubakis I. E. Malliaraki N. Dimoulios P. D. Karmiris K. Castanas E. Kouroumalis E. A. (2004). Decreased Total and Corrected Antioxidant Capacity in Patients with Inflammatory Bowel Disease. Dig. Dis. Sci. 49, 1433–1437. 10.1023/b:ddas.0000042242.22898.d9 15481315

[B16] Lanone S. Bloc S. Foresti R. Almolki A. Taillé C. Callebert J. (2005). Bilirubin Decreases Nos2 Expression via Inhibition of NAD(P)H Oxidase: Implications for protection against Endotoxic Shock in Rats. FASEB j. 19, 1890–1892. 10.1096/fj.04-2368fje 16129699

[B17] Lenicek M. Duricova D. Hradsky O. Dusatkova P. Jiraskova A. Lukas M. (2014). The Relationship between Serum Bilirubin and Crohn's Disease. Inflamm. Bowel Dis. 20, 481–487. 10.1097/01.MIB.0000440817.84251.98 24407487

[B18] Longhi M. S. Vuerich M. Kalbasi A. Kenison J. E. Yeste A. Csizmadia E. (2017). Bilirubin Suppresses Th17 Immunity in Colitis by Upregulating CD39. JCI insight 2. 10.1172/jci.insight.92791 PMC5414551 28469075

[B19] Lu L. Qin A. Huang H. Zhou P. Zhang C. Liu N. (2011). Shikonin Extracted from Medicinal Chinese Herbs Exerts Anti-inflammatory Effect via Proteasome Inhibition. Eur. J. Pharmacol. 658, 242–247. 10.1016/j.ejphar.2011.02.043 21392503 PMC3299007

[B20] Masoodi I. Kochhar R. Dutta U. Vaishnavi C. Prasad K. K. Vaiphei K. (2012). Evaluation of Fecal Myeloperoxidase as a Biomarker of Disease Activity and Severity in Ulcerative Colitis. Dig. Dis. Sci. 57, 1336–1340. 10.1007/s10620-012-2027-5 22350781

[B21] Murphy M. P. (2009). How Mitochondria Produce Reactive Oxygen Species. Biochem. J. 417, 1–13. 10.1042/bj20081386 19061483 PMC2605959

[B22] Naito Y. Takagi T. Yoshikawa T. (2007a). Molecular Fingerprints of Neutrophil-dependent Oxidative Stress in Inflammatory Bowel Disease. J. Gastroenterol. 42, 787–798. 10.1007/s00535-007-2096-y 17940831

[B23] Naito Y. Takagi T. Yoshikawa T. (2007b). Neutrophil-dependent Oxidative Stress in Ulcerative Colitis. J. Clin. Biochem. Nutr. 41, 18–26. 10.3164/jcbn.2007003 18392100 PMC2274988

[B24] Papatheodoridis G. V. Hamilton M. Mistry P. K. Davidson B. Rolles K. Burroughs A. K. (1998). Ulcerative Colitis Has an Aggressive Course after Orthotopic Liver Transplantation for Primary Sclerosing Cholangitis. Gut 43, 639–644. 10.1136/gut.43.5.639 9824344 PMC1727300

[B25] Pisoschi A. M. Pop A. (2015). The Role of Antioxidants in the Chemistry of Oxidative Stress: A Review. Eur. J. Med. Chem. 97, 55–74. 10.1016/j.ejmech.2015.04.040 25942353

[B26] Png C. W. Lindén S. K. Gilshenan K. S. Zoetendal E. G. McSweeney C. S. Sly L. I. (2010). Mucolytic Bacteria with Increased Prevalence in IBD Mucosa Augment *In Vitro* Utilization of Mucin by Other Bacteria. Am. J. Gastroenterol. 105, 2420–2428. 10.1038/ajg.2010.281 20648002

[B27] Reifen R. Nissenkorn A. Matas Z. Bujanover Y. (2004). 5-ASA and Lycopene Decrease the Oxidative Stress and Inflammation Induced by Iron in Rats with Colitis. J. Gastroenterol. 39, 514–519. 10.1007/s00535-003-1336-z 15235867

[B28] Roessner A. Kuester D. Malfertheiner P. Schneider-Stock R. (2008). Oxidative Stress in Ulcerative Colitis-Associated Carcinogenesis. Pathol. - Res. Pract. 204, 511–524. 10.1016/j.prp.2008.04.011 18571874

[B29] Salomone F. Li Volti G. Rosso C. Grosso G. Bugianesi E. (2013). Unconjugated Bilirubin, a Potent Endogenous Antioxidant, Is Decreased in Patients with Non-alcoholic Steatohepatitis and Advanced Fibrosis. J. Gastroenterol. Hepatol. 28, 1202–1208. 10.1111/jgh.12155 23425054

[B30] Sands B. E. (2015). Biomarkers of Inflammation in Inflammatory Bowel Disease. Gastroenterol. 149, 1275–1285. e1272. 10.1053/j.gastro.2015.07.003 26166315

[B31] Sedghi S. Fields J. Z. Klamut M. Urban G. Durkin M. Winship D. (1993). Increased Production of Luminol Enhanced Chemiluminescence by the Inflamed Colonic Mucosa in Patients with Ulcerative Colitis. Gut 34, 1191–1197. 10.1136/gut.34.9.1191 8406152 PMC1375452

[B32] Serafini M. Del Rio D. (2004). Understanding the Association between Dietary Antioxidants, Redox Status and Disease: Is the Total Antioxidant Capacity the Right Tool? Redox Rep. 9, 145–152. 10.1179/135100004225004814 15327744

[B33] Simmonds N. J. Allen R. E. Stevens T. R. J. Niall R. Van Someren M. Blake D. R. (1992). Chemiluminescence Assay of Mucosal Reactive Oxygen Metabolites in Inflammatory Bowel Disease. Gastroenterol. 103, 186–196. 10.1016/0016-5085(92)91112-h 1319369

[B34] Tian S. Li J. Li R. Liu Z. Dong W. (2018). Decreased Serum Bilirubin Levels and Increased Uric Acid Levels Are Associated with Ulcerative Colitis. Med. Sci. Monit. 24, 6298–6304. 10.12659/msm.909692 30196310 PMC6142868

[B35] Tiribelli C. Ostrow J. D. (2005). Intestinal flora and Bilirubin. J. Hepatol. 42, 170–172. 10.1016/j.jhep.2004.12.002 15664240

[B36] Ungaro R. Mehandru S. Allen P. B. Peyrin-Biroulet L. Colombel J.-F. (2017). Ulcerative Colitis. The Lancet 389, 1756–1770. 10.1016/s0140-6736(16)32126-2 PMC6487890 27914657

[B37] Vasavda C. Kothari R. Malla A. P. Tokhunts R. Lin A. Ji M. (2019). Bilirubin Links Heme Metabolism to Neuroprotection by Scavenging Superoxide. Cel Chem. Biol. 26, 1450–1460. e1457. 10.1016/j.chembiol.2019.07.006 PMC6893848 31353321

[B38] Vítek L. Carey M. C. (2003). Enterohepatic Cycling of Bilirubin as a Cause of 'black' Pigment Gallstones in Adult Life. Eur. J. Clin. Invest. 33, 799–810. 10.1046/j.1365-2362.2003.01214.x 12925040

[B39] Wang W. W. Smith D. L. H. Zucker S. D. (2004). Bilirubin Inhibits iNOS Expression and NO Production in Response to Endotoxin in Rats. Hepatol. 40, 424–433. 10.1002/hep.20334 15368447

[B40] Xu C.-T. Meng S. Y. Pan B. R. (2004). Drug Therapy for Ulcerative Colitis. Wjg 10, 2311–2317. 10.3748/wjg.v10.i16.2311 15285010 PMC4576279

[B41] Zhao X. Li L. Li X. Li J. Wang D. Zhang H. (2019). The Relationship between Serum Bilirubin and Inflammatory Bowel Disease. Mediators Inflamm. 2019, 5256460. 10.1155/2019/5256460 31148945 PMC6501121

[B42] Zhu H. Li Y. R. (2012). Oxidative Stress and Redox Signaling Mechanisms of Inflammatory Bowel Disease: Updated Experimental and Clinical Evidence. Exp. Biol. Med. (Maywood) 237, 474–480. 10.1258/ebm.2011.011358 22442342

[B43] Zucker S. D. Vogel M. E. Kindel T. L. Smith D. L. H. Idelman G. Avissar U. (2015). Bilirubin Prevents Acute DSS-Induced Colitis by Inhibiting Leukocyte Infiltration and Suppressing Upregulation of Inducible Nitric Oxide Synthase. Am. J. Physiol. Gastrointestinal Liver Physiol. 309, G841–G854. 10.1152/ajpgi.00149.2014 PMC4652140 26381705

